# Siloxane Matrix Molecular Weight Influences the Properties of Nanocomposites Based on Metal Complexes and Dielectric Elastomer

**DOI:** 10.3390/ma14123352

**Published:** 2021-06-17

**Authors:** Alina Soroceanu, George T. Stiubianu

**Affiliations:** Inorganic Polymers Department, Institute of Macromolecular Chemistry, Petru Poni Iasi, Aleea Grigore Ghica Voda 41A, 700487 Iasi, Romania

**Keywords:** polymer composites, siloxane molecular weight, dielectric permittivity, dielectric elastomer, energy harvesting

## Abstract

Siloxane-based elastomers are some of the most sought-after materials for the construction of actuators and equipment for energy harvesting devices. This article focuses on changes of the mechanical (breaking stress, breaking strain, Young’s modulus) and dielectric properties for elastomers prepared with silicones, induced by the variation of molecular weight of the matrix, with three different silicone polymers having 60,000 g/mol, 150,000 g/mol, and 450,000 g/mol (from GPC measurements). Multiple siloxane elastomers were crosslinked with methyltriacetoxysilane using the sol-gel route. The dielectric permittivity values of the elastomers were also enhanced with two different complex structures containing siloxane bond and *3d* transition metals as filler materials for polydimethylsiloxane polymers with various molecular weights. The dielectric spectroscopy tests demonstrated a small decrease (5%) for the values of the dielectric permittivity in relation to increased molecular weight of the siloxane polymer, both for samples prepared with pure polymer and for samples with metal complexes. The samples of nanocomposites showed a >50% increase of dielectric permittivity values relative to samples prepared of pure siloxane elastomer. The thermal tests demonstrated that the nanocomposites retained thermal stability similar with samples prepared of pure siloxane elastomer. The behavior under controlled conditions of humidity showed a trend of increased water vapor sorption with increasing molecular weight but an overall hydrophobic stable character of nanocomposites.

## 1. Introduction

Over the last three decades, there has been an increased focus on the development of materials that can work as actuators or energy harvesting devices [1,2]. In these applications, mechanical energy from kinetic processes such as human walking, wind, or sea waves is converted to direct electrical current. While demonstration installations have been built for such technologies, it is still necessary to develop methods for material optimization with a focus on specific applications [3]. Studies conducted in this area have highlighted the fact that natural muscles generate linear motion, while electric motors in industrial robots, consumers, and generators in power plants generate circular motion [4,5]. A device that acts as an artificial muscle or energy harvesting device should provide the same linear motion. The materials used in such a device should have high energy densities and large strains, in a similar fashion with natural muscles in the animal kingdom. Polymer elastomers are a clear choice for such devices, as they not only have high energy densities and large strains but are also lightweight, low noise, and low cost. Silicone-based dielectric elastomer polymers are soft polymeric materials that bring together the properties required for actuation and energy harvesting: large energy density, fast response times, operation in air, long lifetimes, and low Young’s modulus as well as high value for breaking strain [6,7]. Additionally, silicone-based materials have the advantage of chemical and structural stability for large variations of environmental and technical parameters such as temperature, humidity, and electrical frequency of applied current [8].

Silicones have the disadvantages of low permittivity [9], and this leads to the increased activation voltages required for reasonable actuation effects. The literature presents the use of multiple types of fillers capable of improving the permittivity of dielectric polymer materials by using one of the following materials: ceramic particles such as titanium dioxide, barium titanate, magnesium niobate; [10,11,12,13,14], conductive particles such as carbon allotropes, metal complexes and copper-phthalocyanine/polyaniline [15,16]; and conjugated polymers such as poly(3-hexyltiophene), polyaniline, polythiophene [17], that can be introduced in the siloxane matrix by blending or as nanoparticles [11,18,19,20,21,22,23,24].

From this approach, the present work is focused on the changes of properties important for dielectric elastomers of polydimethylsiloxane polymers with multiple molecular weights and two types of dielectric constant-enhancing fillers. This study furthers previous work on the influence of the molecular weight of silicones on the properties of materials such as phase separation morphology, oxygen permeability, and mechanical properties [25]; transparency and mechanical properties of silicone block copolymers [26]; and as a matrix for explosives [27]; with a focus on mechanical and dielectric properties, which for dielectric siloxanes are important not only for electric circuits and cables but also for actuators and energy harvesting devices built with silicone dielectric elastomers [8]. The fillers used for the dielectric elastomers are distinct from materials used as fillers by other researchers due to presence of the organosiloxane chain in the complex that provides compatibility with the silicone matrix. To our knowledge there are few studies on using Schiff base metal complexes as filler for siloxanes [28,29]. With structures built around nitrogen atoms and ease of synthesis, the Schiff bases are desirable chelating agents [29]. The ease of synthesis allows the tuning of the properties of interest of the Schiff bases for targeted applications in biomimetic chemistry [30], analytical chemistry [31], materials chemistry [32], and catalysis [33,34,35,36], with efficient catalysis of click reaction in aqueous media [37], cyclic carbonates [38], spin crossover complexes and materials for magnetic recording [39,40,41], and single crystal materials with large dielectric constant [42]. The ability of Schiff bases to coordinate the metal within the polymer-based macromolecules provides the functionality required for sensors and actuators [43,44].

In a previous work, we introduced as fillers for polydimethylsiloxane (PDMS) matrix (Mn = 60,000) multiple metal complexes that have in their structure both the siloxane bond and metal such as manganese, iron, and chromium [15]. The samples of nanocomposites made with PDMS and metallic clusters were compared with samples made just with PDMS elastomer. The significant improvement of both the breaking strain and dielectric permittivity values and dielectric properties overall when such structured fillers are used for nanocomposites was evident [15]. Building on this research, in this study we present new data for nanocomposite siloxane-based materials with two metal complexes containing siloxane bonded iron (C1) and chromium (C2) complexes with ligands containing siloxane spacers. Additionally, the fillers used have transition metals bonded by coordinative bonds in the filler structure, with a direct positive effect on the value of dielectric constant when used in the formulation of a dielectric elastomer. Such structures are soluble in common organic solvents, with molecular-level dispersion of the metal complex in the matrix.

The described approach presents the additional benefits of the ability to process the elastomers as uniform films and of the use of room temperature crosslinking. In this paper, the versatility of silicone polymers with room temperature crosslinking [45,46,47] was used for preparation of nanocomposites with improved values for dielectric constant, while preserving the excellent stretchability of elastomers based on silicones, with breaking strain >500% relative to initial length of sample. Siloxane compounds with different fillers were extensively characterized in terms of their mechanical, dielectric, morphological, surface, and thermal properties with the purpose of being used for wave energy recovering. This paper is focused on the changes induced in these properties by changes in the molecular weight of the polymer matrix for improved properties as dielectric elastomers.

## 2. Materials and Methods

### 2.1. Materials

Iron (C1) and chromium (C2) complexes with ligands containing siloxane spacers were synthesized for further use as filler particles following a previously developed procedure [34] and described in Supplementary Materials (ESI). The polydimethylsiloxane-α,ω-diolmatrices for nanocomposites were synthesized according to the already described procedure [15,48,49]. Molecular weights of the obtained polymers were estimated on the basis of GPC analysis as 60,000 g/mol and ĐM = 1.38 (RS1), 150,000 g/mol and ĐM = 1.2 (RS2), 450,000 g/mol and ĐM = 1.4 (RS3).

Methyltriacetoxysilane (MTS) was prepared and purified in-house using a proprietary technique (purity > 98%, b.p. = 94–95 °C, d420 = 1.20) [50].

Dibuthyltin dilaurate (DBTDL) from Sigma-Aldrich, f.p. = 113 °C, d_4_^20^ = 1.066, was used as received.

### 2.2. Methods

#### 2.2.1. FT-IR Spectroscopy

Fourier transform infrared (FT-IR) spectra were recorded using a Vertex 70 FT-IR spectrometer (Bruker, Erlangen, Germany). Analyses were performed in the transmission mode in the 400–4000 cm^−1^ range, at room temperature, with a resolution of 2 cm^−1^ and accumulation of 32 scans. The samples were incorporated in dry KBr and processed as pellets in order to be analyzed.

#### 2.2.2. Energy-Dispersive X-ray Fluorescence 

The presence and ratio of metals within the composite films were evidenced and their ratio was estimated using an energy-dispersive X-ray fluorescence (EDXRF) system EX-2600 X-Calibur SDD (Xenemetrix, Migdal HaEmek, Israel). The quantitative analysis by this method is subjected to limitations and implies suitable calibration. On the other hand, it is a fast and non-destructive method, which can use very small amounts of the sample.

#### 2.2.3. Scanning Electron Microscopy (SEM)

SEM images were taken using a Quanta 200 scanning electron microscope (FEI company, Brno, Czech Republic) in a low vacuum mode, while the energy-dispersive X-ray spectroscopy (EDX) system available on the equipment was used for elemental mapping.

#### 2.2.4. Mechanical Strain Testing

Equipment TIRA test 2161 (Maschinenbau GmbH Ravenstein, Germany) was used for stress–strain mechanical tests. For each sample an extension rate of 20 mm/min until breaking point was applied at ambient temperature.

#### 2.2.5. Dielectric Spectroscopy

Dielectric spectroscopy was performed on a Novocontrol setup operating in the frequency range of 10^−1^–10^6^ Hz (Concept 40 GmbH) with an ALPHA analyzer (Novocontrol, Montabaur, Germany) and a Quatro temperature controller (Novocontrol, Montabaur, Germany). Samples having uniform thickness in the 0.7–1 mm range were placed between gold-plated round electrodes, the upper electrode having a 20 mm diameter. The metal complexes used as fillers were also tested: these were grounded into fine powders in an agate mortar and then pressed with a 10 ton force press, forming pills with 13 mm diameter and 1 mm thickness.

We recorded for all samples electric breakdown strength (EBD) by measuring with a Trek installation, consisting of different parts: high-speed high-voltage power amplifier (Tektronix P6015A) (Tektronix, Inc., Beaverton, OR, USA), function generator (AFG 3000 wave generator) (Tektronix, Inc., Beaverton, OR, USA), and oscilloscope (Tektronix, Inc., Beaverton, OR, USA). The smallest value out of three recorded was taken into consideration for each sample. The breakdown voltage tests were performed on a homemade setup based on international standards. The samples were equilibrated with the atmospheric humidity by keeping for 24 h before measurements and then were placed before measurements between aluminum discs with 2.5 cm diameter. The voltage applied was increased with 500 V·s^−1^ at a temperature of 20 °C and relative humidity RH~70–72%.

#### 2.2.6. Differential Scanning Calorimetry (DSC)

DSC measurements were conducted with a DSC 200 F3 Maia (Netzsch, Selb, Germany). A mass of 10 mg of each sample was heated in aluminum crucibles with pierced and shut lids. A heating rate of 10 °C min^−1^ was applied, and nitrogen was used as an inert atmosphere (flow rate 50 mL min^−1^). The device was calibrated with indium, according to standard procedures.

#### 2.2.7. The Dynamic Water Vapor Sorption (DVS)

DVS capacity of the samples was determined in the relative humidity (RH) range 0–90% using the fully automated gravimetric analyzer IGAsorp, produced by Hiden Analytical, Warrington (UK).

#### 2.2.8. Gel Permeation Chromatography (GPC)

The molecular mass of the silicone polymers was determined by gel permeation chromatography (GPC) in CHCl_3_ using the equipment PL-EMD 950 chromatograph/evaporative mass detector (Polymer Laboratories, Amherst, MA, USA).

## 3. Results

### 3.1. Preparation of Nanocomposite Materials and Films Formation 

The first step consisted of the dissolving in chloroform (10 wt% solution) of the metal complex (Supplementary Materials, Figure S1). It was followed by mixing a known weighted amount of siloxane with the desired amount of metal complex solution—either C1 (iron complex) or C2 (chromium complex). To the mixture were added measured amounts of methyltriacetoxysilane (MTS) (for crosslinking) and catalyst dibuthyltindilaurate (1% wt relative to siloxane weight) (Table 1).

**Scheme 1:**
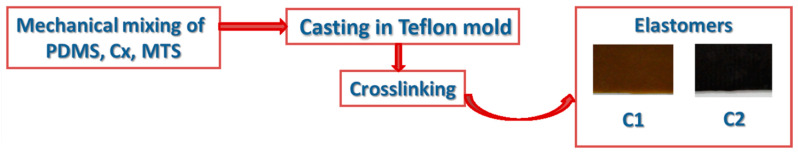
Steps for preparation of composite films.

The next step involved casting the formulation for crosslinking and aging (14 days) in the laboratory environment (Scheme 1), and afterwards it was extremely easy to peel off the films from the surface of the molds.

### 3.2. Structure of Coordination Metal Complex Fillers for Nanocomposites

**Scheme 2:**
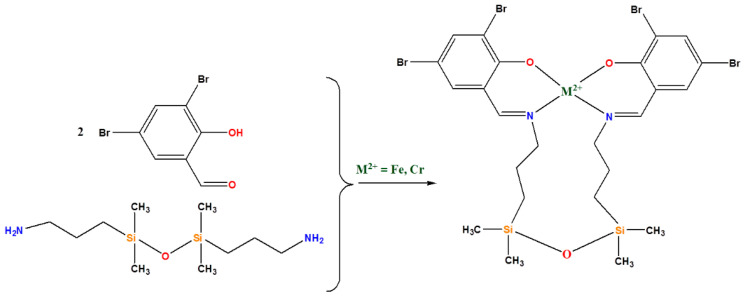
Structures of the metal complexes used as fillers.

The Schiff base ligands used in this work were prepared by condensation reaction between 1,3-bis(3-aminopropyl)tetramethyldisiloxane (AP_0_) and 3,5-dibromosalicylaldehyde (Scheme 2) and were then used for preparation of complexes with iron and chromium. These represented the filler material for silicone polymers due to their structures containing both highly hydrophobic bis(propyl)tetramethyldisiloxane moiety and polar azomethine-metal complex group.

### 3.3. SEM Analysis of the Samples

**Figure 1:**
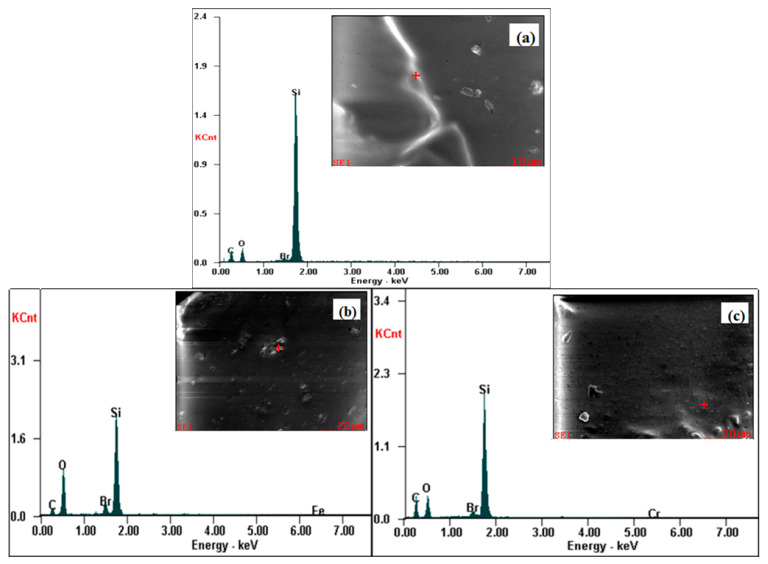
SEM and EDX data for samples: (**a**) RS2; (**b**) C1S2 15%; (**c**) C2S2 15%.

The siloxane molecular chains have freedom of rotation around the Si–O bonds with the methyl groups at the Si atom out of the plane of Si–O bond. The siloxane molecules are also characterized by low surface energy and therefore, when the elastomer forms by crosslinking, these molecules tend to cover the surface of elastomer films usually with a thin layer of siloxane polymer [24], and the true morphology and composition of the material is hidden. Because of this, the samples were first frozen in liquid nitrogen, manually fractured, with the fractured surface revealing the true morphology of the elastomer, and afterwards subsequently analyzed by SEM (Figure 1a–c).

### 3.4. Dielectric Properties of the Samples

**Figure 2:**
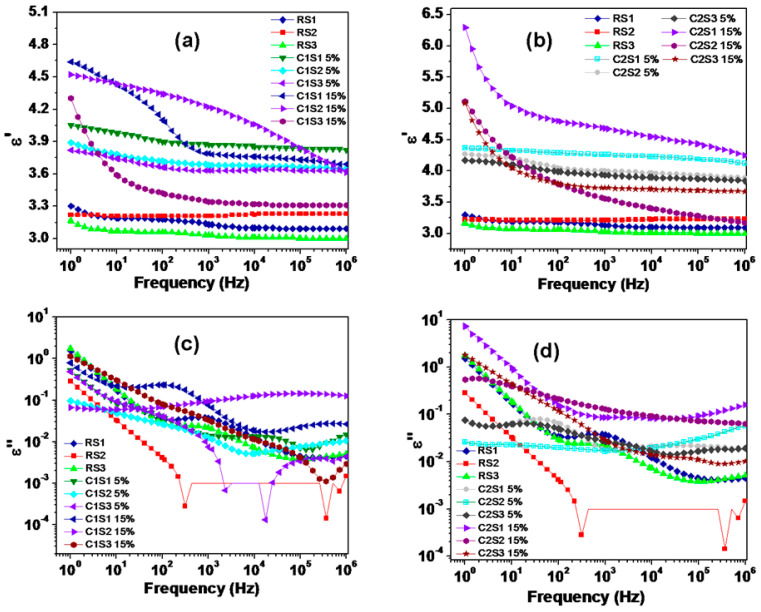
Dielectric properties for samples tested: (**a**) relative permittivity for composites prepared with iron complex (C1); (**b**) relative permittivity for composites prepared with chromium complex (C2); (**c**) loss factor for composites prepared with iron complex (C1); (**d**) loss factor for composites prepared with chromium complex (C2).

Dielectric spectroscopy was performed on the samples in the frequency range 10^−1^–10^6^ Hz, and the dielectric properties (relative permittivity ε’ and loss factor ε”) are shown in Figure 2.

### 3.5. Electromechanical Sensitivity of the Tested Samples

The Young’s modulus values influence the behavior of the elastomers in actuation and energy harvesting, according to the Equation (1) for the compression strain [6]:

(1) $$\frac{\Delta z}{z_{0}}=\frac{p}{Y}=\left(\frac{1}{Y}\right)\cdot \varepsilon^{,}\cdot \varepsilon_{0}\cdot E^{2}=\beta \;\cdot \;\varepsilon_{0}\;\cdot E^{2}$$

where Δ*z* is the decrease of film thickness due to the electric field, *z*_0_ is the initial thickness of the film, *p* is the Maxwell stress, *Y* is the Young’s modulus of the elastomer, *ε’* is its relative permittivity, *ε_0_* is the permittivity of vacuum, *β* is the electromechanical sensitivity, *E* is the electric field strength.

**Figure 3:**
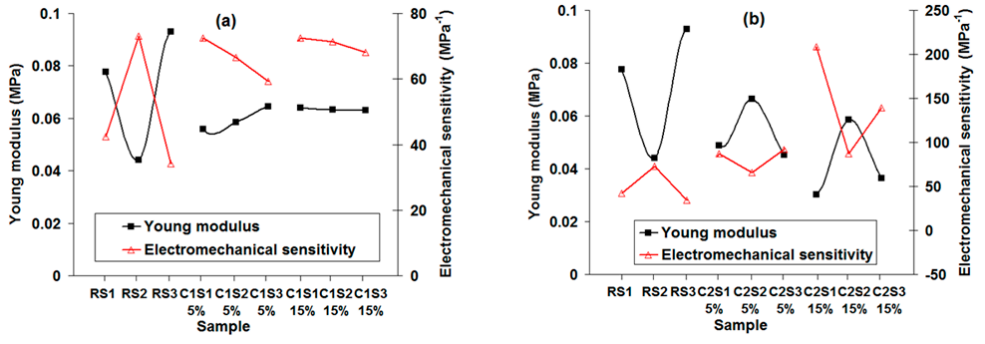
Electromechanical sensitivity of the samples compared with the Young’s modulus values: (**a**) samples with complex C1; (**b**) samples with complex C2.

The variation of Young’s modulus and electromechanical sensitivity as function of the molecular weight of the polymer matrix and the content of metal complex are presented in Figure 3.

The dielectric elastomer films with thickness <1 mm were tested for electrical breakdown strength (EBD) (Table 2) and show there is an increase of EBD value when the molecular weight of the polymer is increasing for samples with lower content of metal complex (0% and 5%), while samples with larger content (15%) of metal complex behave differently (Supplementary Materials, Figure S2).

### 3.6. Dynamic Water Vapour Sorption Results

**Figure 4:**
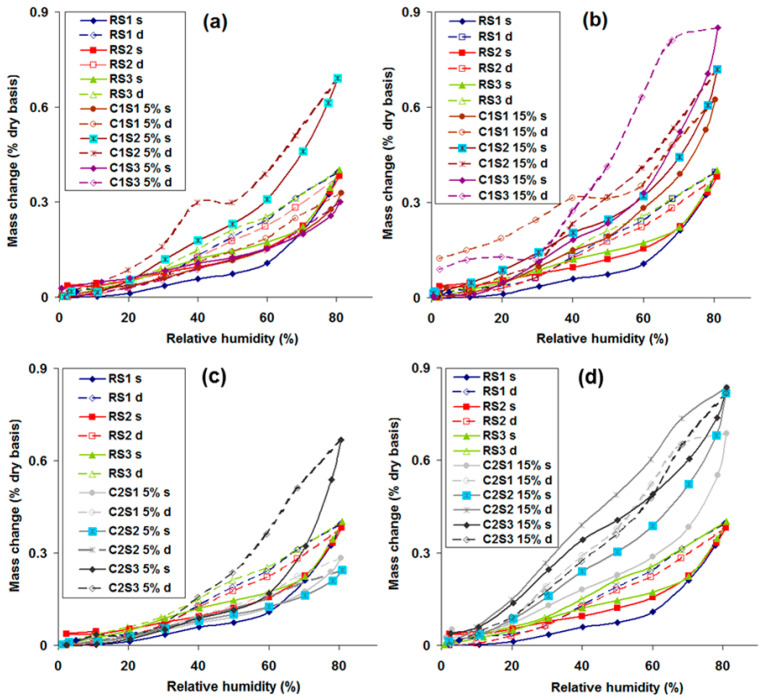
Dynamic vapor sorption of the samples: (**a**) with low content of complex C1 (5%); (**b**) with high content of complex C1 (15%); (**c**) with low content of complex C2 (5%); (**d**) with high content of complex C2 (15%).

The behavior of the dielectric elastomers in the presence of water vapor is important for the construction of devices for actuation and energy harvesting. In Figure 4, there are presented the sorption/desorption isotherms for water vapors at 25 °C, with total water uptake per sample mass in relation to the environmental relative humidity for the samples containing the metal complexes.

## 4. Discussion

The composition of the samples was verified using XRF, and the results obtained (Supplementary Materials, Figure S3) confirm the presence of elemental silicon in all samples and also the presence of metal in nanocomposite samples (PDMS-metal cluster). The peak assigned to rhodium is due to the composition of the electrode, and the peak for argon is due to residual molecules of gas from air in the sample chamber.

In addition to rare aggregates appearing in all samples of silica particles [49], composite samples show high density of micrometer-sized aggregates (Figure 1). Although the metal complexes contain siloxane segment aggregates within the non-polar environment, the introduction of these types of complexes leads to the formation of a larger number of micrometer-sized particles that contain the complex and silica condensed together, according to EDX analysis (Figure 1). As the molecular weight of the polymer matrix increases, the microparticles of metal complex and silica are more thoroughly inserted in the matrix, with a more homogeneous aspect of the cryofractured samples.

**Figure 5:**
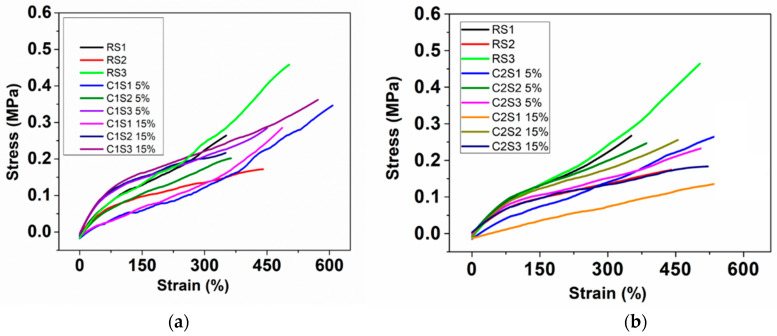
Stress–strain results for the tested samples: (**a**) variation of properties with siloxane molecular weight for samples with iron (C1); (**b**) variation of properties with siloxane molecular weight for samples with chromium (C2).

The mechanical testing of samples was performed with TIRA test 2161, Maschinenbau GmbH Ravenstein. The tests were performed at room temperature, with the strain rate for each sample tested of 20 mm/min, and the data were charted on stress–strain charts (Figure 5).

In the tensile strength tests (Figure 5) performed at room temperature, it is visible that with increasing molecular weight for pure crosslinked polydimethylsiloxane (samples RS1, RS2, and RS3) there is also an increase of breaking stress and strain values. When complex C1 with iron is introduced in the composition of the samples, there is a decrease of the breaking stress values but not of the breaking strain, indicating the metal complex particles act as softening filler for the siloxane matrix (Figure 5a).

The behavior of samples prepared with complex C1 is similar with that of reference samples containing pure siloxane RS1, RS2, and RS3. However, the breaking strain increases by over 50% for sample C1S1 5% in comparison with sample RS1, as the metal complex is compatible with the matrix due to the siloxane bond in its structure, and the particles containing the metal complex probably act also as crosslinking centers. An increased content of complex C1 in samples (15% wt relative to siloxane) does not lead to improved mechanical properties in comparison with the samples with lower complex content (5% wt relative to siloxane), and this indicates that the softening action of the complex containing particles is counteracted by the stress concentrator behavior of these particles.

When coordination complex C2 with chromium is used, there are different effects depending on the molecular weight of the siloxane polymer matrix used for preparation of nanocomposites (Figure 5b). For samples prepared with small molecular weight siloxane RS1, there is a decrease of the breaking stress values accompanied by an increase of the breaking strain, indicating that this coordination metal complex acts as a softening filler that is homogeneously mixed with the siloxane matrix. For samples prepared with medium molecular weight siloxane polymer RS2 were recorded lower breaking strain values and increased breaking stress values as the sample had larger content of metal complex: from 0% wt to 5% wt and then to 15% wt, indicating that in this case there is a small reinforcing filler effect of the particles containing the metal complex, since in this case the particles are slightly smaller (1–3 μm) in comparison with the particles formed in samples with small molecular weight siloxane (3–5 μm).

For the samples prepared with metal complex C2 as filler and large molecular weight siloxane as matrix (RS3, M = 450,000 g/mol), the addition of the metal complex leads only to a decrease of the value of breaking stress; thus, the particles of this type of metal complex act as simple bulk filler for the elastomer and at larger concentration (15% wt) as a stress concentrator, leading to even smaller value for breaking stress.

Overall, the samples prepared with the large molecular weight siloxane (RS3) show the best mechanical properties among samples prepared with pure siloxane. The introduction of metal complexes does not markedly improve the mechanical properties values since the particles containing metal complex act generally as either softener or bulking filler. Only samples prepared with the iron complex C1S1 5 wt. % and C1S3 15 wt. % showed improved values for the breaking strain when compared with reference samples prepared with pure siloxane elastomer.

For samples prepared with pure polydimethylsiloxanes (RS1, RS2, RS3), there is visible an increase of the breaking strain and breaking stress (Supplementary Materials, Figure S4a,b), along with increasing molecular weight of the samples from 6 × 10^4^ to 45 × 10^4^ g/mol. This is due to the fact that elastomers with longer siloxane chains are more flexible and have longer segments between the crosslinking points.

However, for elastomers containing metal complex as fillers, there is not a clear evolution of the breaking stress and breaking stress values. Contrary to films prepared from pure polydimethylsiloxanes, the introduction of metal complex particles leads to a marked improvement of the mechanical properties of samples prepared with low molecular weight RS1, proving that in this case the metal complex particles act as reinforcing filler. The other samples of composite elastomers with RS2 and RS3 show no improvement of mechanical properties, and there is even a decrease of breaking stress values for samples prepared with long chain polymer RS3; thus, the metal complex particles act as bulk filler in terms of mechanical properties.

The behavior of dielectric elastomers in actuation and energy harvesting is determined by their dielectric properties (Figure 2). The metal complexes used for the preparation of the samples of dielectric elastomers presented in this paper contain in their structure short siloxane bonds that are compatible with the matrix made from PDMS, while the metal core present in the complex leads to improved values for the relative permittivity of the resulting dielectric elastomers.

For dielectric spectroscopy tests, the powdery metal complexes were pressed manually into 13 mm diameter and 1 mm thick pellets. The iron complex C1 has a value for relative permittivity of 7.52 × 10^6^, and the loss factor is 2 × 10^7^ at 100 Hz, with conductivity of 2 × 10^−5^ S/cm. For the complex with chromium C2, the relative permittivity is 7, the loss factor is 0.4 at 10^0^ Hz, and conductivity values are 10^−13^–10^−8^ S/cm in the frequency domain tested. The values of relative permittivity are larger than those specific for siloxanes (~3). Therefore, if the metal complex particles do not percolate in the PDMS matrix so as to avoid conductivity of current, then such coordination complexes could lead to better properties, such as dielectric permittivity and electromechanical sensitivity for the prepared dielectric elastomers.

The reference samples prepared with pure crosslinked siloxane show values of the dielectric constant between 3 and 3.3, in the range known for siloxanes (Figure 2a). The sample with average molecular weight siloxane (RS2) has the best stability of this parameter over the range of frequencies tested: 10^0^–10^6^ Hz, and it also has the lowest value for the loss factor among the three reference samples (Figure 2c).

For the samples containing iron complex C1 as filler (5 wt.% relative to siloxane), the value of the dielectric constant changes: the dielectric constant decreases with increasing value of the siloxane molecular weight (C1S1 5%–C1S2 5%–C1S3 5%), and also there is a gentle decrease of this parameter value with increasing frequency from 1 to 10^6^ Hz (Figure 2a). The same behavior is recorded for the loss factor, but the largest value for the loss factor is just 0.5 (Figure 2c). The increase of the content of metal complex in nanocomposite to 15 wt. % leads to increased values for the dielectric constant up to 4.7 for sample C1S1 15 wt. % but with a steeper decline of this value when sweeping the frequency range, and larger values were also recorded for the loss factor (Figure 2c).

When complex C2 with chromium is used (Figure 2b) in the composition of the elastomers, there is a similar behavior of samples, with increasing molecular weight of the siloxane used as in the case of complex C1. The effect of the metal complex is more pronounced since the samples present larger values of the dielectric constant when compared with samples prepared with complex C1 (Figure 2a), both for low content (5 wt. %) and high content of metal complex (15 wt. %) (up to 6.3 for sample C2S1 15%). However, only samples with low content of metal complex have acceptable values for the loss factor (ε” < 0.5), while an increase of the amount of complex C2 in samples leads to loss factor values that are too large for the material to be considered dielectric—namely, ε” > 1 (Figure 2d).

The values of the dielectric constant decrease when the molecular weight increases since this increases the free volume in the siloxane network (Supplementary Materials, Figure S5). In addition, the dielectric constant increases with the increase in the content of metal complex in the sample of dielectric elastomer; this effect is the largest for the samples prepared with the polymer S1 of low molecular weight.

When elastomers are intended for use as dielectric materials, the electromechanical sensitivity is an important variable of the elastic energy density, as it is proportional to the main ratio ε’ε_0_/Y from Equation (1) [19,51], and for calculations, ε’ was measured at 1 Hz, and Y was measured at breaking strain. The change of Young’s modulus values in relation to the electromechanical sensitivity (β), which is another important parameter for actuation and energy harvesting, and therefore the variation of these two parameters for various samples are presented together in the same charts (Figure 3a,b), shows improved electromechanical sensitivity in comparison with pure siloxane samples due to the presence of siloxane bond in the structure of each of the complexes’ ligands, with excellent compatibility between the metal complex and the silicone matrix in the absence of surfactant.

As expected, there is an increase in the value of Young’s modulus with increasing molecular weight of the siloxane (samples RS1, RS2, RS3). A similar behavior is also recorded for samples with low content of iron complex (Figure 3a), but the other samples with metal complex in their composition do not have a clear pattern of evolution for this parameter. The samples with metal complex have lower values for Young’s modulus than the reference samples, showing that metal complexes act as softening or bulk filler for the siloxanes.

Samples with complex C2 also show increased values for β when compared with the reference samples with pure crosslinked siloxane (Figure 3b), but there is no specific pattern for the evolution of this parameter. The samples with high content (15%) of C2 (C2S1 15%, C2S2 15%, C2S3 15%) present much larger values for β than the other samples; however, these three samples also have large values for loss factor ε” (Figure 2d), which is not conducive for application in devices.

The electromechanical sensitivity (β) values slightly decrease with increasing molecular weight for reference samples. The addition of complex C1 leads to clearly larger values for β, and the values for this parameter decrease with increasing molecular weight of the siloxane matrix, both for samples with low content (5%) and high content (15%) of metal complex (Figure 3a). Additionally, the increase in the content of metal complex from 5 wt. % to 15 wt. % leads to samples with increased values of β.

For samples prepared with complex C1 15%, although EBD increases with molecular weight, it is still lower than the values for RS of corresponding molecular weight. Samples prepared with complex C2 15% not only have lower EBD values than the references RS, but these values decrease with the increase of molecular weight. This behavior for samples with a large content of metal complex is due to the aggregation of particles, forming conductive pathways through the polydimethylsiloxane matrix. However, the variation of the EBD values for each sample demonstrates this is related to sample homogeneity, and future work will be focused on films prepared in a space with controlled atmosphere and possibly without the use of solvents.

DSC analysis (Supplementary Materials, Figures S6 and S7) in the range −150 up to +150 °C indicates a glass transition (Tg) between −121–−123 °C on the second heating run. This demonstrates that the complete embedding of metal complex particles inside the siloxane network as the filler does not change the Tg of siloxane. For the reference samples prepared with pure siloxane, there is a small increase in the temperature of the endothermic peak attributed to the melting of crystalline phase from −43 (for RS1) to −41 °C (for RS3), due to higher crosslinking degree (Supplementary Materials, Figure S7a–c). The same behavior is exhibited by the exothermic peak corresponding to crystallization, with the temperature increasing from −73 (for RS1) to −71 °C (for RS3). The peak corresponding to melting (−42 °C) is less intense than the one corresponding to crystallization (−72 °C), indicating no significant development of crystalline phase during cooling, due to strong crosslinking of siloxane. A similar behavior is observed for DSC curves of samples with metal complexes C1 and C2, but temperatures are decreased to −75 (for RS1) and −73 °C (for RS3) (Supplementary Materials, Figures S6d–f and S7d–f). This behavior indicates that the particles with metal complex act as softening filler in the siloxane materials. The molecular weight of the siloxane is the most important factor influencing the thermal behavior of the samples.

From DSC data (Supplementary Materials, Figure S6 and S7), it can be seen that the samples have Tg values in the range −121–−123 °C. The heat capacity value of this transition was used for calculating the crosslinking density values (Equation (2)):

(2) $$\rho_{c}=-\frac{\left(C_{p}^{i}-C_{p}^{0}\right)}{C_{p}^{0}}$$

where *C^i^_p_* and *C*^0^*_p_* are the heat capacities of the polymer network at a given crosslinking density and that of the non-crosslinked polymer, respectively [52,53].

The degree of crosslinking increases with the decreasing molecular weight of the siloxane used for reference samples (Table 3).

There is a similar variation when metal complexes are introduced in the sample—a decrease of the crosslinking degree values with increasing molecular weight of the siloxane used for that sample. This behavior is due to the fact that siloxanes with higher molecular weight (RS2 and RS3) have larger free volume between the siloxane chains. Therefore, the particles with metal complex, while acting as reinforcing filler for small molecular weight siloxane RS1, act as bulking/softening filler for siloxanes with high molecular weight.

The shape of the isotherms for reference samples prepared with pure siloxane (RS1, RS2, and RS3) is similar to those with uptake of 0.3 wt. % relative to the dry mass of the sample at 80% relative humidity. This behavior confirms the hydrophobic character of the samples.

When small content of metal complexes (5%) is introduced in the composition of the sample (Figure 4a,c), there is a smaller water vapor uptake in comparison with pure siloxane reference samples for samples with polymers RS1 and RS2, while samples with large molecular weight siloxane RS3 and metal complex show large water uptake. The particles of metal complexes cover the pores in siloxanes with small and medium molecular weight, thus leaving little space for water vapor uptake. The samples prepared with high molecular weight siloxane RS3 have high free volume between the siloxane chains, and the introduction of metal complexes that form particles together with silica cannot close all the pores and also form hydrophilic surfaces for the condensation of water vapors inside the siloxane matrix pores, thus leading to increased final value of water uptake.

The samples prepared with a content of 15% metal complex (Figure 4b,d) show an increased water vapor uptake amount in direct relation to the siloxane molecular weight mass, as the presence of numerous particles with metal complex increase the porosity of the samples and thus open the way for increased water vapor sorption. All samples have maximum water vapor sorption values at or below 0.85% weight dry basis, and thus the hydrophobic character of pure siloxane samples is preserved.

The weight of the samples reverts to that recorded initially for most samples when the sorption–desorption cycle ends, and this is proof of the moisture stability of the samples at room temperature. There are samples, such as C1S1 15%, C1S2 15%, and C1S3 15%, with the end point of the cycle different than the starting point, as the large content of metal complex particles leads to sorption–desorption hysteresis and smaller desorption speed. Thus, a large cut-off time on the order of hours would be required in order to allow for the weight of the samples to return to the initial point on the desorption curve. The moisture sorption isotherms are type IV (according to IUPAC classification). Mesoporous materials with slit-shaped pores [54] have H3 type hysteresis loops and pore condensation, which are visible in the sorption/desorption isotherms.

The values for the maximum mass change due to sorption of water vapors shows that the general trend is an increase of this parameter in step with the increase of the molecular weight of the polydimethylsiloxane used for samples preparation (Supplementary Materials, Figure S8), and a decrease of the mass change is visible once the content of metal complex increases, progressing in the series RS—C1 5%—C2 5%—C1 15%—C2 15%.

## 5. Conclusions

Films of dielectric elastomers were prepared by room temperature condensation with siloxane polymers of different molecular weights and with metal complexes as fillers. SEM microscopy proved that the formation of micrometer-sized particles that contain metal complex and silica resulted from the hydrolysis of methyltriacetoxysilane, distributed uniformly in the polydimethylsiloxane polymers matrix. The mechanical properties (such as breaking strain, breaking strength) of the reference samples prepared with pure polydimethylsiloxanes improved with the increasing molecular weight of the polydimethylsiloxane used for the specific sample from 360% breaking strain at 0.27 MPa for RS1 (M = 60,000) to 510% breaking strain and 0.45 MPa for RS3 (M = 450,000). The introduction of metal complexes in the composition of the samples only increased breaking strain values, as the metal complex particles act as softening bulking filler, while breaking stress was under 0.35 MPa for all samples with metal complex filler. Overall, the materials possessed breaking strains over 300% and Young’s moduli less than 0.1 MPa.

The variation of the molecular weight of the polydimethylsiloxanes did not significantly change the value of the dielectric constant. However, the stability of the value of relative permittivity (ε’) varied with the molecular weight of the polymers: the sample RS2 (prepared with medium molecular weight polydimethylsiloxane) showed constant values for ε’ in all the range of frequencies studied (1–10^6^ Hz), while samples R1 and R3 had a strong variation of ε’ with frequency. With the introduction of the metal complex as filler, the values of the dielectric constant increased by over 100% in comparison with values measured for pure siloxane samples up to ε’ = 4.35 for C2S1 15% at 10^3^ Hz and ε’ = 4.75 for C2S1 15% at 10^3^ Hz. For the samples prepared with metal complex with chromium C2, there was also a large increase of the loss factor; thus, samples prepared with iron complex C1 are most suitable for use in further applications for actuator and energy harvesting devices based on dielectric elastomers [49]. The introduction of metal complexes in the elastomer did not influence in a major way the values of the electromechanical sensitivity β, but this parameter had decreasing values with the increase of the molecular weight of the polydimethylsiloxane used. The values of β, coupled with the ones for loss factor in dielectric measurements, indicate the optimum samples for applications in actuation and energy harvesting are those prepared with iron complex with low and medium molecular weight polydimethylsiloxane C1S1 and C1S2.

The increase of the molecular weight of the polymer led to a slight increase in water vapor sorption, especially for samples with a high content (15%) of metal complex. The values for water vapor sorption for all samples maintained in the band specific for hydrophobic materials with weak interactions between sorbent and sorbed material, with sorbed water of up to 0.85% dry weight of the sample.

The data presented in this paper describe the influence of the variation in molecular weight of a silicone polymer over the mechanical and electric properties and can facilitate the choice of silicones with optimum molecular weight for specific applications as a function of the desired combination of properties: mechanical strain, dielectric constant, strength and conductivity, and water vapor sorption.

## Figures and Tables

**Table 1:** The feed amounts of the precursors for film nanocomposites.

Sample	Composition, % wt					
	PDMS			MTS	Metal Complex **	
	S1	S2	S3			
RS1 *	100	-	-	8	-	-
RS2 *	-	100	-	8	-	-
RS3 *	-	-	100	8	-	-
C1S1 x% **	100	-	-	8	5	15
C1S2 x%	-	100	-	8	5	15
C1S3 x%	-	-	100	8	5	15
C2S1 x%	100	-	-	8	5	15
C2S2 x%	-	100	-	8	5	15
C2S3 x%	-		100	8	5	15

* RS, reference sample, one for each siloxane polymer (S1, S2, S3). ** x%, the amount of metal complex used, as weight percentage of the polydimethylsiloxane matrix.

**Table 2:** Mechanical and dielectric properties of the dielectric elastomers.

Sample	Breaking Stress (MPa)	Stress at 20% Elongation ^a^	Elongation at Break (% Initial Length)	Young’s Modulus at 20% Elongation	ε’ (10 Hz)	ε” (10 Hz)	EBD (MV/m)
RS1 *	0.27	0.0340	352	0.0147	3.19	0.175	21
RS2 *	0.20	0.0278	440	0.0097	3.21	0.138	25
RS3 *	0.46	0.0644	503	0.0490	3.07	0.190	30
C1S1 5%	0.33	0.0655	605	0.0640	3.98	0.088	35
C1S2 5%	0.21	0.0494	363	0.0240	3.78	0.050	39
C1S3 5%	0.29	0.0541	450	0.0201	3.74	0.094	30
C2S1 5%	0.28	0.0450	581	0.0420	4.2	0.065	24
C2S2 5%	0.25	0.0454	385	0.0214	4.33	0.023	27
C2S3 5%	0.23	0.0380	504	0.0180	4.1	0.062	31
C1S1 15%	0.31	0.0350	486	0.0220	4.43	0.218	12
C1S2 15%	0.22	0.0620	351	0.0290	4.44	0.060	21
C1S3 15%	0.36	0.0540	572	0.0181	3.59	0.305	28
C2S1 15%	0.20	0.0130	649	0.0083	5.03	0.970	15
C2S2 15%	0.26	0.0350	455	0.0186	4.23	0.398	11
C2S3 15%	0.19	0.0550	521	0.0458	4.05	0.441	10

* RS, reference sample for each siloxane polymer (S1, S2, S3). ^a^ This is in the elastic range of the prepared dielectric elastomer materials.

**Table 3:** Calculation of crosslinking degree from DSC tests.

Sample	Heat Capacity, *C*_p_ (J∙g^−1^∙K^−1^)	Crosslinking Density, *ρ*_c_ (mol∙cm^−3^) ^a^	Sample	Heat Capacity, *C*_p_ (J∙g^−1^∙K^−1^)	Crosslinking Density, *ρ*_c_ (mol∙cm^−3^) ^a^
PDMS ^b^	0.100	0	C2S2 5%	0.090	0.10
RS1 *	0.013	0.87	C2S3 5%	0.090	0.10
RS2 *	0.056	0.44	C1S1 15%	0.073	0.27
RS3 *	0.095	0.05	C1S2 15%	0.091	0.09
C1S1 5%	0.062	0.38	C1S3 15%	0.090	0.10
C1S2 5%	0.092	0.08	C2S1 15%	0.070	0.30
C1S3 5%	0.097	0.03	C2S2 15%	0.098	0.01
C2S1 5%	0.072	0.28	C2S3 15%	0.097	0.03

* RS, reference sample, for each siloxane polymer (S1, S2, S3). ^a^ Determined using Equation (2). ^b^ PDMS, non-crosslinked polydimethylsiloxane.

## Data Availability

Not applicable.

## Supplementary Materials

The following are available online at https://www.mdpi.com/article/10.3390/ma14123352/s1, Figure S1: FTIR spectra of the synthesized metal complexes: (a) C1; (b) C2, Figure S2: Variation of Electric Breakdown Strength values in relationship with the molecular mass of the siloxane and the type of metal complex used for the sample, Figure S3: XRF analysis for the elastomer composite films: (a) RS1; (b) C1S1 5%, Figure S4: Variation of breaking stress (a) and breaking strain (b) values in relationship with the molecular mass of the siloxane and the type of metal complex used for the sample, Figure S5: Variation of dielectric constant values in relationship with the molecular mass of the siloxane and the type of metal complex used for the sample, Figure S6: DSC results for the dielectric elastomers prepared: (a), (b), (c) reference samples RS1, RS2, RS3; (d), (e), (f) samples with low content (5%) of C1; (g), (h), (i) samples with high content (15%) of C1, Figure S7: DSC results for the dielectric elastomers prepared: (a), (b), (c) reference samples RS1, RS2, RS3; (d), (e), (f) samples with low content (5%) of C2; (g), (h), (i) samples with high content (15%) of C2, Figure S8: Variation of recorded DVS mass change values (as % of dry mass) in relationship with the molecular mass of the siloxane and the type of metal complex used for the sample.
