# Supplementary material for: Siloxane Matrix Molecular Weight Influences the Properties of Nanocomposites Based on Metal Complexes and Dielectric Elastomer
Source: Materials (Basel). 2021 Jun 17;14(12):3352. doi: 10.3390/ma14123352 (PMC8234613; doi:10.3390/ma14123352)
Supplement: Supplementary file 1 [file materials-14-03352-s001.zip › materials-1239879-SI.pdf]

Electronic Supplementary Material

The Effect of Molecular Weight of Siloxane Matrix on the Properties of Metal Complexes Dielectric Elastomer Nanocomposites

Alina Soroceanu[*], George T. Stiubianu[*]

Inorganic Polymers Department, Institute of Macromolecular Chemistry "Petru Poni " Iasi

1. Preparation of metal siloxane complexes

Each metal complex was prepared according with the general pathway described in *Scheme 1.*

1.1. Preparation pathway for iron-based complex

3,5-Dibromosalicylaldehyde (2.021 g) in MeOH/$CHCl_3$ (1:1) (10 mL) was added with a dropping funnel to a solution of 1,3-bis(3-aminopropyl)tetramethyldisiloxane (0.897 g) in MeOH/$CHCl_3$ (1:1) (10 mL) and then heated on oil bath at 70 °C for 3 h. Then was added a solution of $Fe(ClO_4)_2 \cdot H_2O$ (1.019 g) in MeOH (5 mL), and the flask was allowed to stand at room temperature under inert atmosphere. The dark brown odourless powder formed very rapidly and was separated by filtration, washed with methanol and dried in air. Yield 23 %. $C_{24}H_{30}O_3N_2Br_4Fe_1Si_2$ (826.13) (C1): IR (KBr pellet, selected bands): ν_{max} = 2955 (w, C–H stretching in CH_3 from from Si–CH_3), 1611 (s, C=N), 1504 (s, aromatic ring), 1408 (s, CH_2), 1256 (s, Si–CH_3), 1078 (vs, Si–O–Si), 795 (m, Si–CH_3) cm^{-1}.

1.2. Preparation pathway for the chromium-based complex

A solution of 1,3-bis(3-aminopropyl)tetramethyldisiloxane (0.897g) in MeOH/$CHCl_3$ (1:1) (10 mL) was added with a dropping funnel to a solution MeOH/$CHCl_3$ (1:1) (10mL) of 3,5-dibromsalicyl aldehyde (2.021 g) under stirring. The reaction mixture was heated at 70 °C for 3 h and then slowly poured into a solution of $Cr(NO_3)_3 \cdot 9H_2O$ (1.6 g, 4 mmol) in MeOH/$CHCl_3$ (1:1) (10 mL). For homogenization the mixture was stirred at room temperature for 10 min and then allowed to stand undisturbed under inert atmosphere. The violet odourless powder formed after the solution stood undisturbed for 3 days, and was washed with methanol and dried in air. Yield 35.0 %.

[*] Corresponding author. Tel. + 4 0232-217454.
Email address: lazar.alina@icmpp.ro (A. Soroceanu); george.stiubianu@icmpp.ro (G. T. Stiubianu).

$C_{24}H_{32}O_3N_2Br_4Cr_1Si_2$ (909.55) (C2): IR (KBr pellet, selected bands): ν_{max} = 2955 (w, C–H stretching in CH_3 from from Si–CH_3), 1599 (s, C=N), 1502 (m, aromatic ring), 1385 (vs, CH_2), 1254 (m, Si–CH_3), 1059 (s, Si–O–Si), 797 (m, Si–CH_3) cm^{-1}.

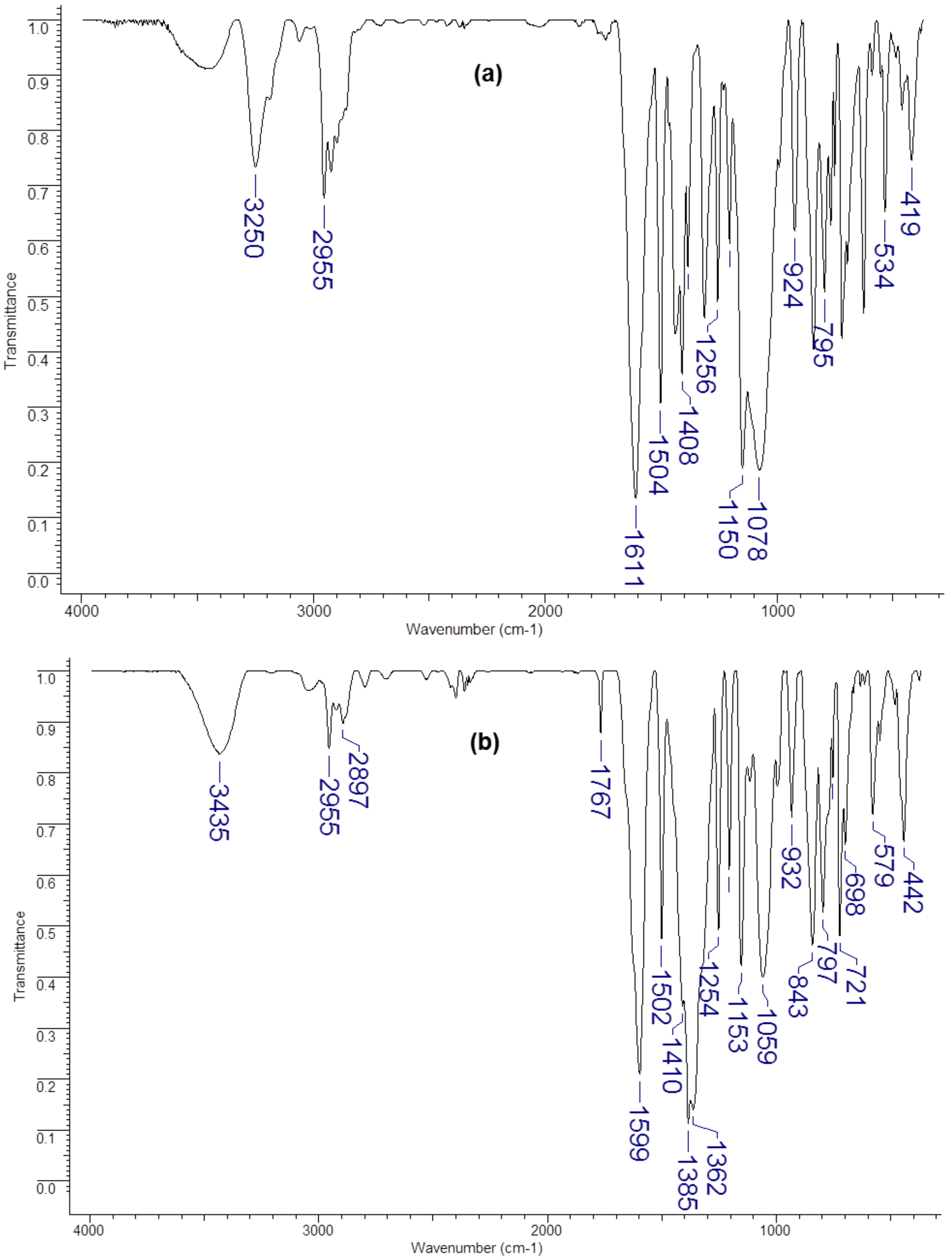

Figure S1. FTIR spectra of the synthesized metal complexes: (a) C1; (b) C2.

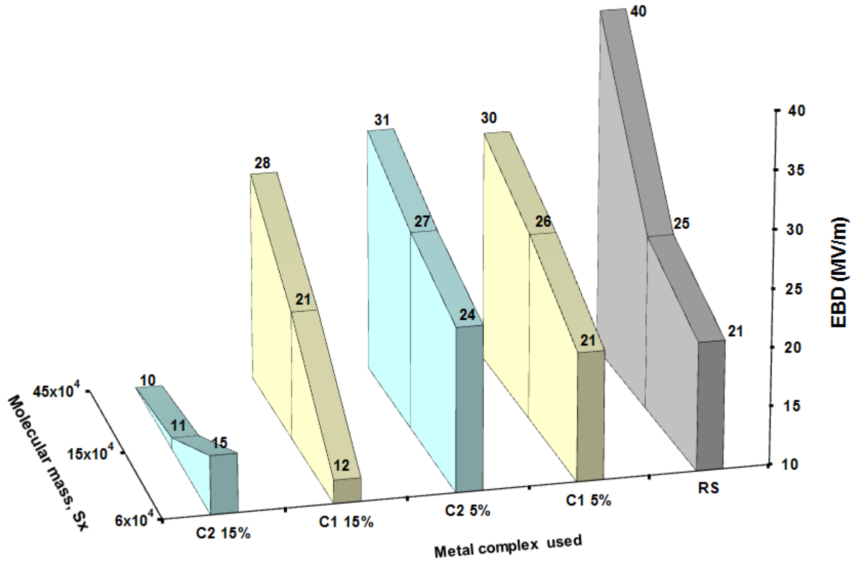

Figure S2. Variation of Electric Breakdown Strength values in relationship with the molecular mass of the siloxane and the type of metal complex used for the sample

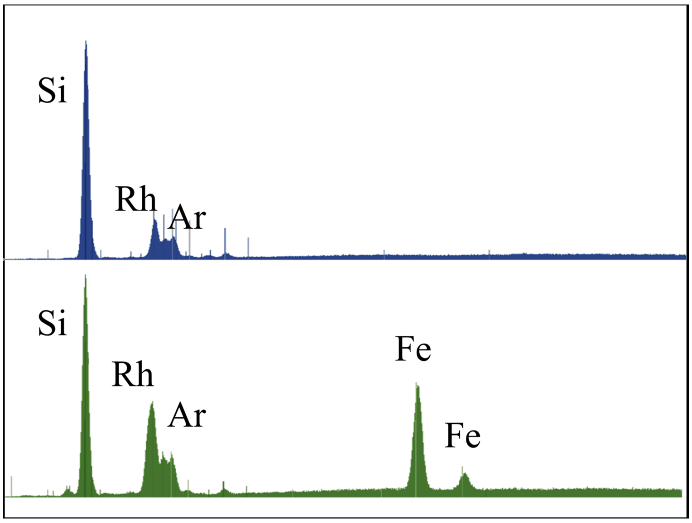

Figure S3. XRF analysis for the elastomer composite films: (a) RS1; (b) C1S1 5%

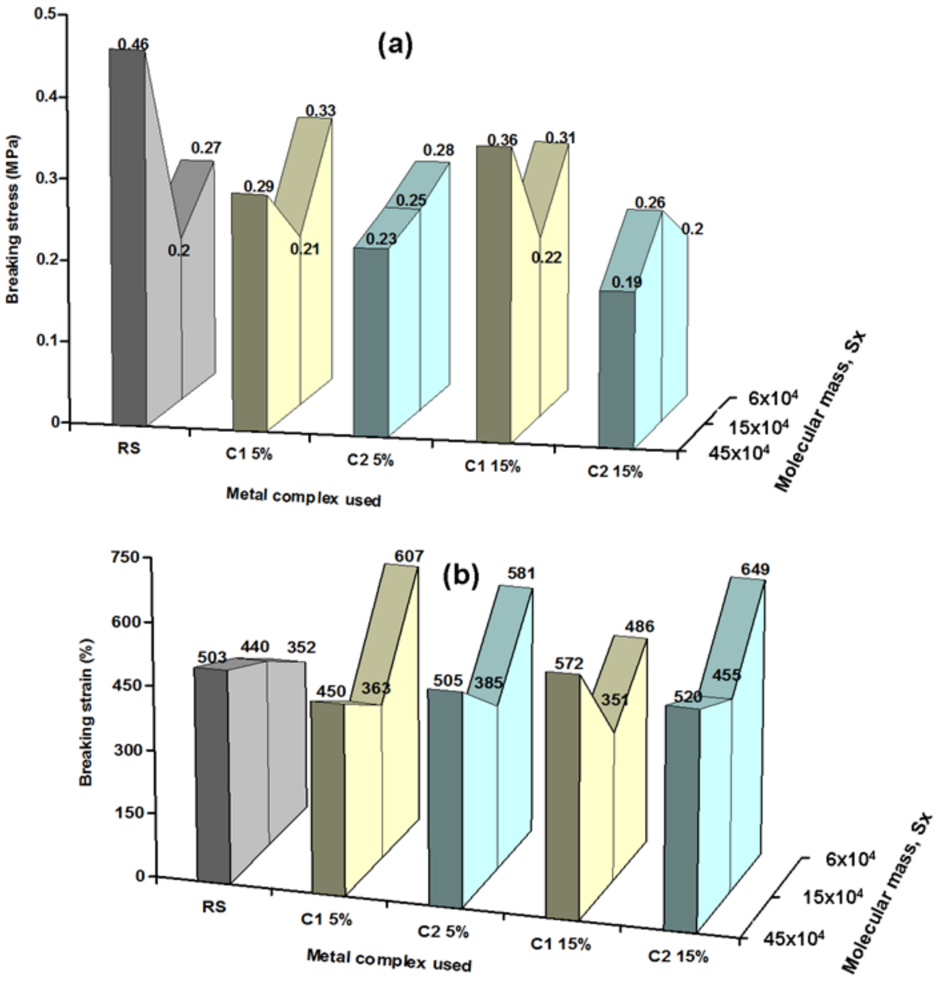

Figure S4. Variation of breaking stress (a) and breaking strain (b) values in relationship with the molecular mass of the siloxane and the type of metal complex used for the sample

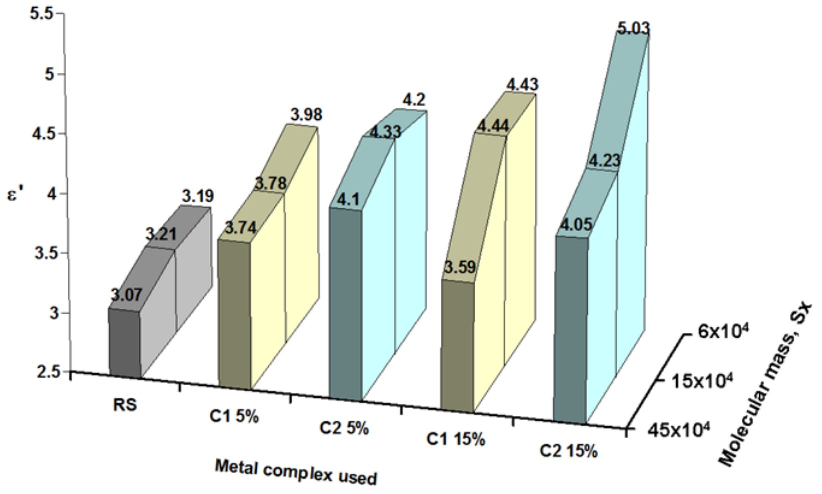

Figure S5. Variation of dielectric constant values in relationship with the molecular mass of the siloxane and the type of metal complex used for the sample

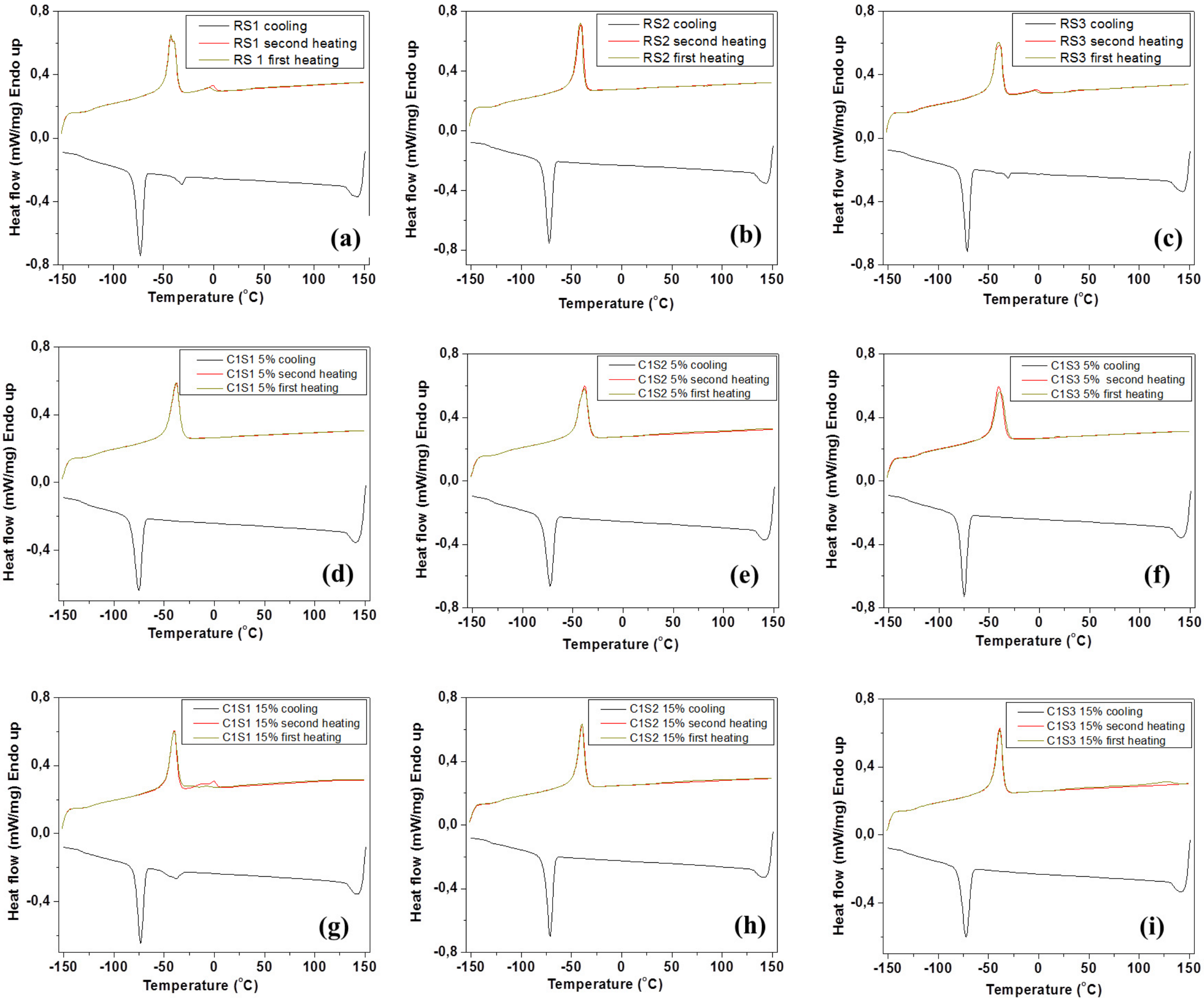

Figure S6. DSC results for the dielectric elastomers prepared: (a), (b), (c) reference samples RS1, RS2, RS3; (d), (e), (f) samples with low content (5%) of C1; (g), (h), (i) samples with high content (15%) of C1

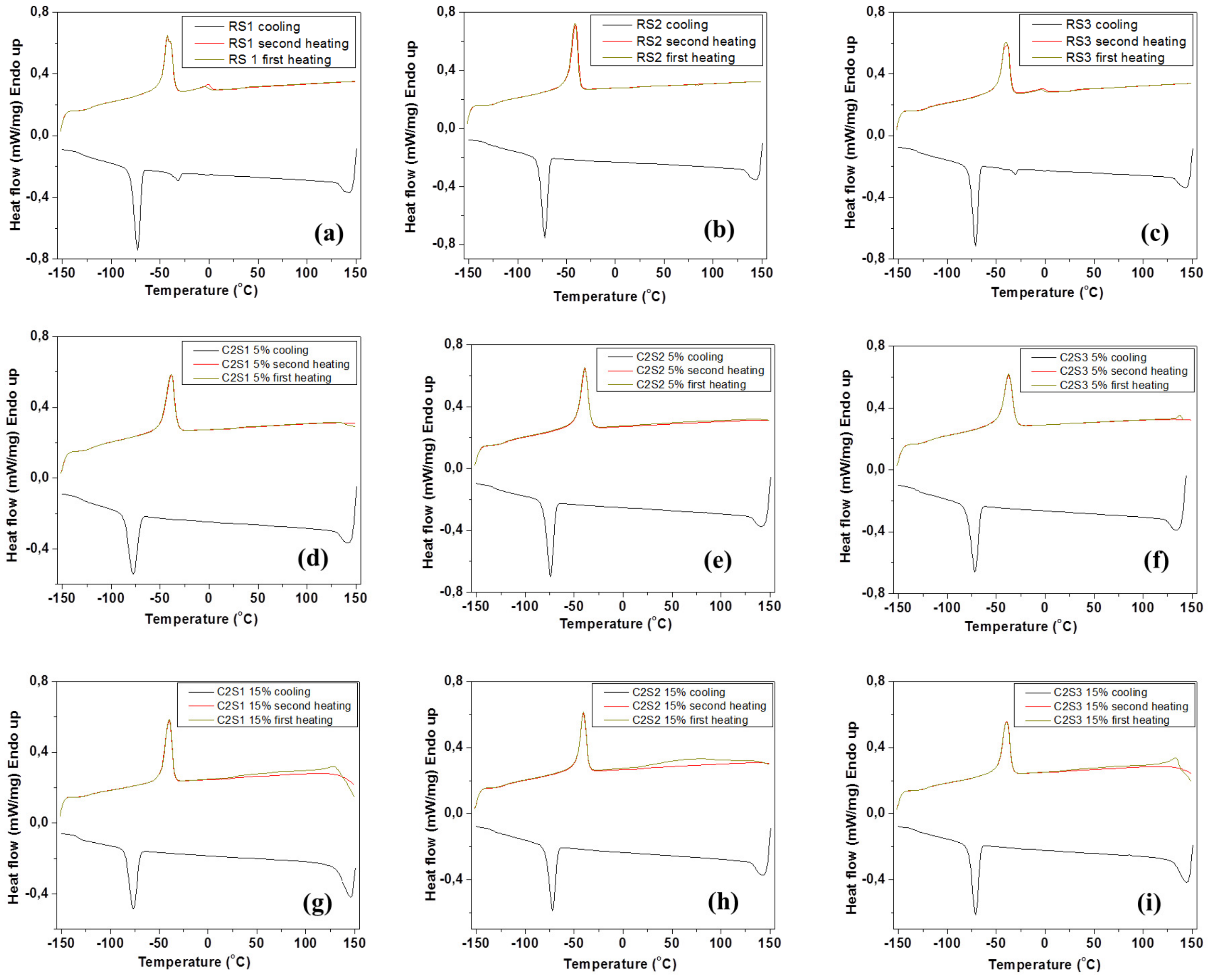

Figure S7. DSC results for the dielectric elastomers prepared: (a), (b), (c) reference samples RS1, RS2, RS3; (d), (e), (f) samples with low content (5%) of C2; (g), (h), (i) samples with high content (15%) of C2

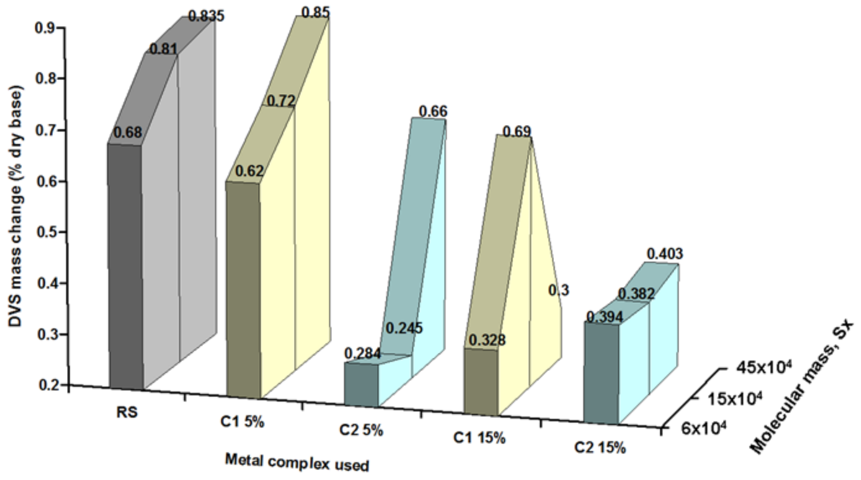

Figure S8. Variation of recorded DVS mass change values (as % of dry mass) in relationship with the molecular mass of the siloxane and the type of metal complex used for the sample
